# Loss of PPARα function promotes epigenetic dysregulation of lipid homeostasis driving ferroptosis and pyroptosis lipotoxicity in metabolic dysfunction associated Steatotic liver disease (MASLD)

**DOI:** 10.3389/fmmed.2023.1283170

**Published:** 2024-01-08

**Authors:** Claudia Theys, Tineke Vanderhaeghen, Evelien Van Dijck, Cedric Peleman, Anne Scheepers, Joe Ibrahim, Ligia Mateiu, Steven Timmermans, Tom Vanden Berghe, Sven M. Francque, Wim Van Hul, Claude Libert, Wim Vanden Berghe

**Affiliations:** ^1^ Protein Chemistry, Proteomics and Epigenetic Signaling (PPES), Department of Biomedical Sciences, University of Antwerp, Antwerp, Belgium; ^2^ Center for Inflammation Research, VIB, Ghent, Belgium; ^3^ Department of Biomedical Molecular Biology, Ghent University, Ghent, Belgium; ^4^ Center of Medical Genetics, University of Antwerp, Antwerp, Belgium; ^5^ Laboratory of Experimental Medicine and Pediatrics, Infla-Med Centre of Excellence, University of Antwerp, Antwerp, Belgium; ^6^ Pathophysiology Lab, Infla-Med Centre of Excellence, Department of Biomedical Sciences, University of Antwerp, Antwerp, Belgium; ^7^ Department of Gastroenterology and Hepatology, Antwerp University Hospital, Edegem, Belgium

**Keywords:** PPARα, MASLD, epigenetics, lipid metabolism, bile acid metabolism, NAFLD, ferroptosis, pyroptosis

## Abstract

Metabolic Dysfunction Associated Steatotic Liver Disease (MASLD) is a growing epidemic with an estimated prevalence of 20%–30% in Europe and the most common cause of chronic liver disease worldwide. The onset and progression of MASLD are orchestrated by an interplay of the metabolic environment with genetic and epigenetic factors. Emerging evidence suggests altered DNA methylation pattern as a major determinant of MASLD pathogenesis coinciding with progressive DNA hypermethylation and gene silencing of the liver-specific nuclear receptor PPARα, a key regulator of lipid metabolism. To investigate how PPARα loss of function contributes to epigenetic dysregulation in MASLD pathology, we studied DNA methylation changes in liver biopsies of WT and hepatocyte-specific PPARα KO mice, following a 6-week CDAHFD (choline-deficient, L-amino acid-defined, high-fat diet) or chow diet. Interestingly, genetic loss of PPARα function in hepatocyte-specific KO mice could be phenocopied by a 6-week CDAHFD diet in WT mice which promotes epigenetic silencing of PPARα function via DNA hypermethylation, similar to MASLD pathology. Remarkably, genetic and lipid diet-induced loss of PPARα function triggers compensatory activation of multiple lipid sensing transcription factors and epigenetic writer-eraser-reader proteins, which promotes the epigenetic transition from lipid metabolic stress towards ferroptosis and pyroptosis lipid hepatoxicity pathways associated with advanced MASLD. In conclusion, we show that PPARα function is essential to support lipid homeostasis and to suppress the epigenetic progression of ferroptosis-pyroptosis lipid damage associated pathways towards MASLD fibrosis.

## 1 Introduction

Non-alcoholic fatty liver disease (NAFLD), recently re-named and re-defined as Metabolic Dysfunction Associated Steatotic Liver Disease (MASLD) (Rinella et al., 2023), is a growing epidemic, paralleling the increase of obesity in western diet consuming countries. MASLD shares, in part, the common pathogenesis of metabolic syndrome including obesity, hyperlipidaemia, insulin resistance, mitochondrial damage, oxidative stress response, and the release of inflammatory cytokines. It has an estimated prevalence of 20%–30% in Europe and is the most common cause of chronic liver disease worldwide (Almeda-Valdés et al., 2009; Loomba and Sanyal, 2013; Younossi et al., 2016). MASLD consists of a spectrum of liver disorders ranging from isolated steatosis to Metabolic Dysfunction Associated Steatohepatitis (MASH) which predisposes patients to progressive fibrosis, cirrhosis and hepatocarcinoma but also extrahepatic diseases, especially cardiovascular diseases (Stepan et al., 2012; Chalasani et al., 2018). Dysregulation of insulin secretion and dyslipidaemia due to obesity and other lifestyle variables are the primary contributors to the establishment of MASLD. Although the prevalence keeps growing, there is still no FDA-approved treatment for MASLD. Therefore, with no drugs available, the mainstay of MASLD management remains lifestyle changes with exercise and dietary modifications (Vilar-Gomez et al., 2015; Romero-Gómez et al., 2017).

The onset and progression of MASLD are orchestrated by an interplay of metabolic environment with genetic and epigenetic (lifestyle, environment) factors (Tiffon, 2018; Juanola et al., 2021). An accumulating body of studies revealed progressive DNA methylation changes across different stages of MASLD pathogenesis, although the underlying mechanisms remain poorly understood (Ahrens et al., 2013; Murphy et al., 2013; Kitamoto et al., 2015; Zeybel et al., 2015; Hardy et al., 2017; Mwinyi et al., 2017; Wegermann et al., 2018; Lai et al., 2020). DNA methylation signatures that can affect gene expression are influenced by environmental and lifestyle experiences such as diet, obesity and physical activity and are reversible (Berger et al., 2009; Alegría-Torres et al., 2011; Tiffon, 2018). Hence, DNA methylation signatures and modifiers in MASLD may provide the basis for developing biomarkers indicating the onset and progression of MASLD and therapeutics for MASLD. More specifically, MASLD patients show global hepatic DNA hypomethylation in parallel with increasing hepatic inflammation grade, disease progression and increased hypermethylation of the promotor sequence of the nuclear receptor peroxisome proliferator-activated receptor-α (PPARα) gene (Francque et al., 2015; Li et al., 2018; Lai et al., 2020). Whether loss of PPARα function is a cause or consequence of epigenetic dysregulation in MASLD pathology requires further investigation.

PPARα is part of the PPAR nuclear receptor family that consists of three isoforms: PPARα, PPARβ and PPARγ. All three isoforms are involved in lipid metabolism, but are most abundantly expressed in liver, skeletal muscle and adipocytes respectively (Bugge and Mandrup, 2010; Francque et al., 2021). Since PPARα is abundantly expressed in the liver, known as a key regulator of lipid metabolism, and downregulated in MASLD patients correlating with the disease stage, several agonists have been therapeutically evaluated over the years (Francque et al., 2015; Kersten and Stienstra, 2017). However, PPARα agonists that only target PPARα failed to show convincingly positive results in clinical trials (Lange et al., 2022). Therefore, research is currently more shifting towards drugs targeting multiple therapeutic targets, i.e., pan-PPAR agonists (e.g., Lanifibranor), but also epigenetic modulators (e.g., vitamine E) (Remely et al., 2017; Boubia et al., 2018). Both have already shown promising results in clinical trials of MASLD patients, suggesting a crucial role for PPAR interplay with epigenetic control mechanisms in the development of MASLD (Remely et al., 2017; Boubia et al., 2018; Sumida and Yoneda, 2018). Indeed, recent papers demonstrate significant demethylation of PPARα target metabolic genes upon activation of PPARα (Ehara et al., 2015; Hashimot et al., 2018; Yuan et al., 2018; Moody et al., 2019). Besides, PPARα interactions with epigenetic enzymes have already been identified in different tissues including liver and colon (Hervouet et al., 2010; Luo et al., 2019; Aibara et al., 2022). To further characterize epigenetic “driver” or “passenger” functions of PPARα in MASLD, we compared genome-wide DNA methylation and transcriptome changes in livers of wild type (WT) and hepatocyte-specific PPARα knock out (KO) mice, receiving control chow diet versus MASLD promoting high fat diet (CDAHFD). Characterisation of genome-wide DNA methylation and gene expression changes might provide new insights in PPARα-dependent (epigenetic driver) versus independent (epigenetic passenger) functions, with potential clinical relevance in precision medicine for disease management and staging of MASLD progression.

## 2 Materials and methods

### 2.1 Mouse model

PPARα KO C57BL/6J (PPARa^fl/fl^ AlbuminCre^Tg/+^) mice and WT C57BL/6J (PPARa^fl/fl^ AlbuminCre^+/+^) mice (IRC-VIB, UGent) were housed in a temperature‐controlled, specific pathogen free (SPF) air‐conditioned animal house with 14/10h light/dark cycles and received food and water *ad libitum*. 7-week old male hepatocyte-specific PPARα KO and WT mice were fed either a chow diet (normal standard diet, containing 9% energy from fat, 58% from carbohydrates, and 33% from protein) or a CDAHFD (choline-deficient L-amino acid defined high-fat diet, A06071302, New Brunswick, NJ United States, containing 62% energy from fat, 20% from carbohydrates, and 18% from protein) for 6 weeks *ad libitum* creating 4 different treatment groups. At 13 weeks, the mice were sacrificed by cervical dislocation after anaesthesia with ketamine and xylazine diluted in phosphate-buffered saline (PBS) (2:2:6). Liver samples were immediately snap frozen and stored at −80°C for further analysis. The animal experiments were approved by the institutional ethics committee for animal welfare of the Faculty of Sciences, Ghent University, Belgium (EC2021-071).

### 2.2 Histology

The liver was excised from euthanized mice and washed in PBS. Excised liver was fixed in 4% paraformaldehyde overnight at 4°C, dehydrated and embedded in paraffin. The excised tissue sections of 5 μm were cut and stained with haematoxylin-eosin (H&E) using standard protocols. Liver sections were also stained using Masson’s Trichrome staining kit (HT15, Sigma-Aldrich), following the manufacturer’s protocol. In short, liver slices were deparaffinized and hydrated using BIDI. Next, the liver slices were treated with the mordant preheated Bouin’s solution for 30 min at 60°C. Then, the slices were washed and stained with Weigert’s iron haematoxylin at room temperature. After washing and rinsing, the liver slices were stained with Biebrich Scarlet-Acid Fuchsin. After rinsing the tissue slices, the slices were put in a Phosphotungstic/phosphomolybdic Acid solution followed by an Aniline Blue solution and 1% acetic acid. Finally, the liver slices were dipped once in 70% ethanol and 90% ethanol followed by washing the liver slices with 100% ethanol and xylene before mounting the liver slices.

### 2.3 RNA extraction

Total RNA was extracted from the livers of the mice after tissue disruption with the TissueRuptor (Qiagen) with the RNeasy kit (Qiagen, 75162), according to the manufacturer’s protocol. Afterwards RNA quantity was determined using Qubit^TM^ RNA Broad Range Assay kit with the aid of the Invitrogen Qubit^TM^ Fluorometer (Thermo Fisher Scientific, United States). The extracted RNA was stored at −80°C until further analysis.

### 2.4 RNA sequencing

Total isolated RNA of the livers of 3 mice of each treatment group were sent to Novogene Leading Edge Genomic Services & Solutions for RNA sequencing analysis on the Novaseq6000 platform. In brief, messenger RNA was purified from total RNA using poly-T oligo-attached magnetic beads. After fragmentation, the first strand cDNA was synthesized using random hexamer primers, followed by the second strand cDNA synthesis and library construction. The library was checked with Qubit and real-time PCR for quantification and bioanalyzer for size distribution detection. Quantified libraries were pooled and sequenced on Illumina platforms, according to effective library concentration and data amount. The quality of the raw sequencing reads was evaluated using FastQC (v0.11.5) (Andrews, 2020) and subsequent alignment to genome reference consortium mouse build 38 (GRCm38) was performed with the STAR (v.2.7.3a) tool (Dobin et al., 2013). Differential gene expression and pathway analysis were performed using DESeq2 R package software (Love et al., 2014) and the Omics Playground tool (v2.8.12) platform, which was also used for further visualisation. RNA sequencing was validated by qPCR and deposited in the NCBI GEO database with accession number GSE238201.

### 2.5 Quantitative polymerase chain reaction (qPCR)

After RNA extraction, total RNA was converted into cDNA with the iScript^TM^ cDNA Synthesis Kit (BioRad, 1708890) according to the manufacturer’s protocol. Next, qPCR analysis was performed using the PowerUp SYBR^TM^ green PCR master mix (Thermo Fisher Scientific, United States) according to the manufacturer’s instructions. In brief, a 20 µL reaction volume mix per sample was prepared containing 10 µL PowerUp SYBR Green Master Mix, 0.4 µM forward and reverse primer (Supplementary Table S1), and nuclease-free water. The following PCR program was applied on the Rotor-Gene Q qPCR machine of Qiagen: 50°C for 2 min, 95°C for 2 min, 40 cycles denaturation (95°C, 15 s) and annealing/extension (60°C, 1 min), and dissociation (60°C–95°C). Each sample was run in triplicate. The mean value of the triplicates was taken to calculate the ΔΔCt-values using GAPDH and YWHAZ as the normalisation genes. PPARα and DNMT1 primer sequences (Supplementary Table S1) were designed by Primer3 and synthesized by Integrated DNA Technologies (IDT, United States). Statistical analysis was carried out using a One-Way ANOVA test with Tukey’s correction for multiple comparisons. *p*-value < 0.05 was considered statistically significant.

### 2.6 Protein extraction and western immunoblot analysis

For western blot analysis, liver tissue was disrupted with the TissueRuptor (Qiagen). Next, cells were lysed in 0.5 mL 1x RIPA lysis buffer (150 mM NaCl, 0.1% Triton x-100, 0.1% SDS, 50 mM Tris-HCl pH 8 supplemented with protease inhibitor cocktail (Sigma-Aldrich, Germany)) on ice for 15 min. Afterwards cells were briefly sonicated and centrifugated at 13,000 rpm for 15 min at 4°C. Next, supernatant with soluble protein extract was transferred to new Eppendorf tubes and used for protein quantification with Pierce™ BCA Protein Assay Kit (Thermo Fisher Scientific, United States). After protein extraction, SDS-PAGE was performed to separate proteins on a 6%–12% gradient Bis-Tris gel. First, samples were mixed with Laemmli buffer (Biorad, United States) and 50 mM 1,4- dithiothreitol (DTT) and then heated at 70°C for 10 min to denaturate the protein. Afterwards, both the samples and protein ladder (BenchMark™ Protein Ladder, Thermo Fisher Scientific, United States) were loaded on the Bis-Tris gel at a protein concentration of 10 µg/well (PPARα, DNMT1, NRF2), 20µg/well (ACSM2A, SLC27A2, CYP7B1, CPT1A) or 100µg/well (Caspase-1 and NLRP3). Electrophoresis was performed in a Mini-PROTEAN Tetra Cell System (Biorad, United States) using a high molecular weight buffer (100 mM MOPS, 100 mM Tris, 0.2% SDS, 2 mM EDTA, 5 mM sodium bisulphite). Afterwards, the proteins were transferred to pre-wet nitrocellulose membranes (Cytiva, United States) for 1 h at 4°C on 250 mA. After blocking the membranes in 5% milk/TBST blocking buffer for 1 h at room temperature, the primary antibodies anti-PPARα (Abcam, #ab126285), anti-DNMT1 (Imgenex, #60B1220.2), anti-NLRP3 (Bio-connect, #AG-20B-0014-C100), anti-Caspase-1 (Bio-connect, AG-20B-0048-C100), anti-NRF2 (Proteintech, #16396-1-AP), anti-CYP7B1 (ProteinTech, #24889-1-AP) and anti-ACSM2A (ProteinTech, #22862-1-AP) were diluted 1:1000; anti-SLC27A2 (ProteinTech, #14048-1-AP) was diluted 1:2000; and anti-CPT1A (ProteinTech, #15184-1-AP) was diltuded 1:4000 in the blocking buffer and incubated overnight at 4°C. The next day, membranes were washed three times with TBST and incubated with HRP-conjugated anti-rabbit secondary antibody (PPARα, DNMT1 and NRF2) or HRP-conjugated anti-mouse secondary antibody (NLRP3 and Caspase-1) diluted in blocking buffer (1:2000) for 1 h at room temperature. Anti-GAPDH antibody (Bioké #5174S, diluted 1:1000) in blocking buffer was used as loading control. Protein detection was performed on the Amarshan imager 680 (Cytiva, United States) using SuperSignal™ West Pico PLUS Chemiluminescent Substrate (Thermo Fisher Scientific, 34577) and quantified using ImageJ software. Statistical analysis was carried out using a One-Way ANOVA test with Tukey’s correction for multiple comparisons. *p*-value < 0.05 was considered statistically significant.

### 2.7 Lipid peroxidation-MDA assay

Liver tissue was disrupted with the TissueLyser II (Qiagen) on 20Hz for 5 min at 4°C in 1 mL PBS. Afterwards 100 µL of the tissue lysate was pipetted in a 96 well plate for MDA quantification and the remaining sample was used for further protein quantification with the Pierce™ BCA Protein Assay Kit (Thermo Fisher Scientific, United States) according to the manufacturer’s protocol. At the same time, a 1:2 serial dilution of 1,1,3,3-tetramethoxypropane (0–20 µM) in MiliQ was made to form a standard curve of MDA under acidic conditions. Subsequently, a working solutions consisting of 0.5 mg N-methyl phenyl indol (NMPI), 0.2 mL Acetonnitrile and 0.08 mL Methanol was added per 100 µL of sample or standard. Afterwards, 75 µL of 37% chloric acid was added to the reaction and the samples were incubated for 45min at 70°C. Next, the reaction was stopped by a centrifugation for 10min at 15000rpm at 4°C and the amount of carbocyanine dye formed during this reaction of MDA with NMPI, was measured at 595 nm using the 2103 EnVision™ Multilabel Plate Reader (Perkin Elmer, United States). The final concentration of MDA was further corrected for protein concentration. Statistical analysis was carried out using a One-Way ANOVA test with Dunnett’s correction for multiple comparisons. *p*-value < 0.05 was considered statistically significant.

### 2.8 Methylation analysis

Whole-genome methylation profiling was performed on the livers of 3 mice of each treatment group using the Infinium Mouse Methylation BeadChip array (Illumina, San Diego, CA, United States) at the Centre for Medical Genetics (Antwerp University Hospital (UZA), University of Antwerp). Genomic DNA (gDNA) was extracted from the livers using the Dneasy Blood & Tissue Kit (Qiagen, 69504, Courtaboeuf, France) according to the manufacturer’s protocol. DNA concentration and purity were determined by the Qubit 4 Fluorometer (Thermo Fisher Scientific, Q33238). Next, 750 ng DNA was bisulphite converted with the EZ DNA Methylation Kit (Zymo Research, D5001/D5002, Irvine, CA, United States) according to the manufacturer’s instructions. Successful bisulphite conversion was confirmed by PCR with the PyroMark PCR kit (Qiagen) in a region of the Line1 gene (Supplementary Table S2). The resulting PCR products were run on a 2% agarose gel. This converted DNA was then further hybridized with the Illumina Infinium Mouse Methylation BeadChip (Illumina, San Diego, CA, United States) according to the manufacturer’s instructions. In brief, converted DNA was amplified overnight and fragmented enzymatically. Precipitated DNA was resuspended in hybridisation buffer and dispended onto the BeadChips. The hybridisation procedure was performed at 48°C overnight using an Illumina Hybridisation oven. After hybridisation, free DNA was washed away, and single nucleotide extension followed by fluorescent readout was performed. The BeadChips were imaged using an Illumina iScan (Illumina, San Diego, CA, United States). The platform interrogates more than 285,000 methylation sites per sample at single-nucleotide resolution. Annotations for the interrogated sites were taken from Illumina’s BeadChip array manifest based on genome build mm10. Raw intensity data from IDAT files was read and processed in R (v. 4.2.0) via the Enmix package and beta values were normalised with the Enmix D method (Xu et al., 2016). Data pre-processing consisted of masking probes with poor design, control probes, and non-cg and non-ch probes. Detection *p*-values were inferred using SeSAMe’s pOOBAH (*p*-value with out-of-band array hybridization) algorithm. Probes with detection *p*-values > 0.01 or more than 10% of NA values were filtered out. No samples had more than 10% missing values, thus all were considered for further analysis. Further probe-type bias adjustment was applied with the Regression on Correlated Probes method (Niu et al., 2016). The difference in signal intensity between the two-colour channels (dye bias correction) was corrected for using a flexible exponential-normal mixture distribution model. Background correction was done using the Out-Of-Band algorithm. To identify significantly differentially methylated CpGs between the different groups of mice, the Wilcoxon rank-sum test with a Bonferroni correction (*p* < 0.01) for the total amount of CpGs in the Mouse Methylation BeadChip was used. Further Metascape pathway analysis of genes with a delta beta (DB) > |0.1| and FDR <0.05 was performed with the online Metascape Web tool (Zhou et al., 2019). Methylation data was deposited in the NCBI GEO database with accession number GSE238173.

### 2.9 Pyrosequencing analysis

Pyrosequencing was used to validate methylation of the RETSAT and Eci1 promotor identified by BeadChip analysis. The sequences of the promotor region of the RETSAT and Eci1 gene were retrieved from the Ensemble website (http://genome.ucsc.edu/). Primers were designed based on this sequence and the PyroMark Assay Design Software 2.0.2. (Qiagen) (Supplementary Table S1). Genomic DNA (gDNA) was extracted from the livers using the Dneasy Blood & Tissue Kit (Qiagen, 69504, Courtaboeuf, France) according to the manufacturer’s protocol. DNA concentration and purity was determined by the Qubit 4 Fluorometer (Thermo Fisher Scientific, Q33238). Next, 750 ng DNA was bisulphite converted with the EZ DNA Methylation Kit (Zymo Research, D5001/D5002, Irvine, CA, United States) according to the manufacturer’s instructions. Successful bisulphite conversion was confirmed by PCR with the PyroMark PCR kit (Qiagen) in a region of the Line1 gene (Supplementary Table S2). The resulting PCR products were run on a 2% agarose gel. After successful bisulphite conversion, a PCR was performed with the PyroMark PCR kit (Qiagen) and a forward and biotinylated reverse primer specific to a cytosine in the promotor region of the RETSAT and Eci1 gene. Afterwards 20 µL of this biotinylated product was further used for pyrosequencing using the PyroMark Q24 system (Qiagen) and PyroMark Q24 advanced reagent kit (Qiagen) in combination with a sequencing primer covering 1 CpG according to the manufacturer’s protocol. Analysis was performed using the Pyromark Q24 Advanced software (version 3.0) for detection and quantification of methylation patterns in the target regions. The only values that were reported to be technically reliable by the PyroMark Q24 Software 2.0.8 (Qiagen) were used for statistical analysis. The One-Way ANOVA was performed to assess differential methylation of RETSAT and Eci1 gene between different treatment groups.

## 3 Results

### 3.1 Hepatocyte-specific PPARα KO and high fat diet disrupt bile and fatty acid metabolism and promote MASLD/MASH like gene expression signatures and histopathology features

**FIGURE 1:**
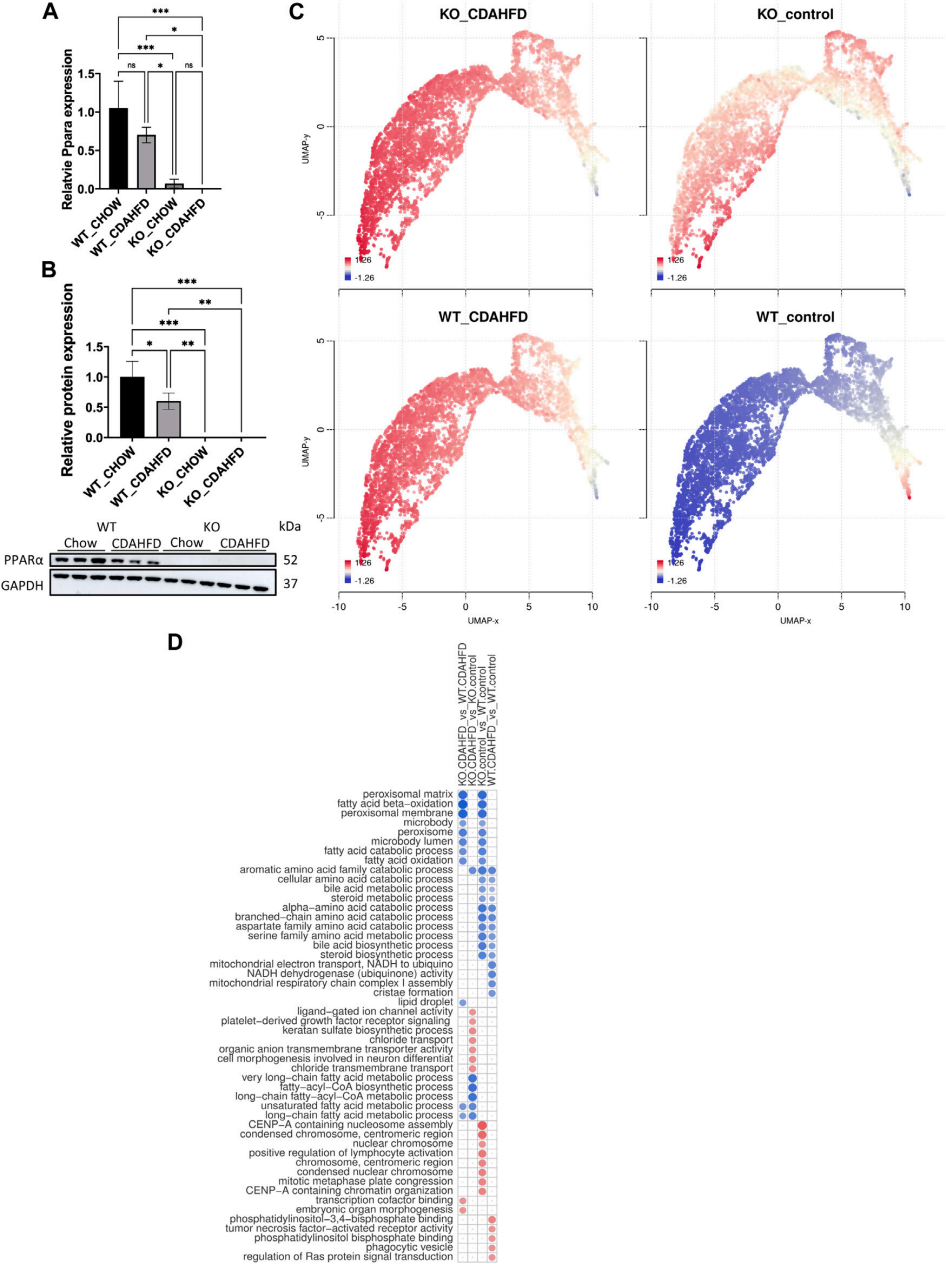
**(A)** Relative *Ppara* mRNA expression in PPARα WT and KO mice after a 6-week chow or CDAHFD. **(B)** Western blot detection and quantification of PPARα and GAPDH expression levels after a 6-week chow or CDAHFD in WT and KO mice. Data are plotted as the mean ± s.d., *n* = 3 biologically independent replicates. (*p < 0.05, **p < 0.01 ***p < 0.001) **(C)** UMAP representation of gene clustering based on geneset co-expression in PPARα WT and KO mice after a 6-week chow or CDAHFD **(D)** GO activation matrix representation of pathway enrichment analysis of significantly up- or downregulated pathways in both comparisons of KO mice versus WT mice on a chow or CDAHFD respectively. The size of the circles in the GO activation matrix corresponds to their relative activation, and are colored according to their upregulation (red) or downregulation (blue) in the contrast profile (meta.q < 0.05).

PPARα, a key player of lipid metabolism and energy homeostasis, is typically downregulated in the livers of MASLD patients (Francque et al., 2015). To characterize the functional role of PPARα in MASLD development, we studied a hepatocyte-specific PPARα KO mouse model following a 6-week CDAHFD (HFD) to simulate liver pathological properties of a prolonged western diet. First, lack of PPARα expression in PPARα KO liver samples was confirmed at the RNA and protein level by qPCR and western blot analysis, respectively (Figures 1A, B). QPCR and western analysis clearly confirm lack of significant PPARα expression in PPARα KO liver samples as expected (some background *Ppara* mRNA residual transcription may originate from traces of non-hepatocytes (mainly from stellate cells) present in the liver biopsies (Gonzalez-Sanchez et al., 2017)). Interestingly, RNAseq transcriptome profiling revealed high similarities in transcription profiles of PPARα KO mice on chow diet versus WT mice following 6-week CDAHFD diet. This suggests that a CDAHFD partially phenocopies loss of hepatocyte function of PPARα, closely resembling a genetic PPARα KO approach (Figure 1C; Supplementary Figure S1). Along the same line, GO gene set enrichment analysis confirms mitochondrial dysfunctions due to multiple changes in bile and fatty acid metabolism, amino acid catabolism and inflammation, in response to CDAHFD and upon genetic PPARα KO or combinations thereof (Figure 1D).

**FIGURE 2:**
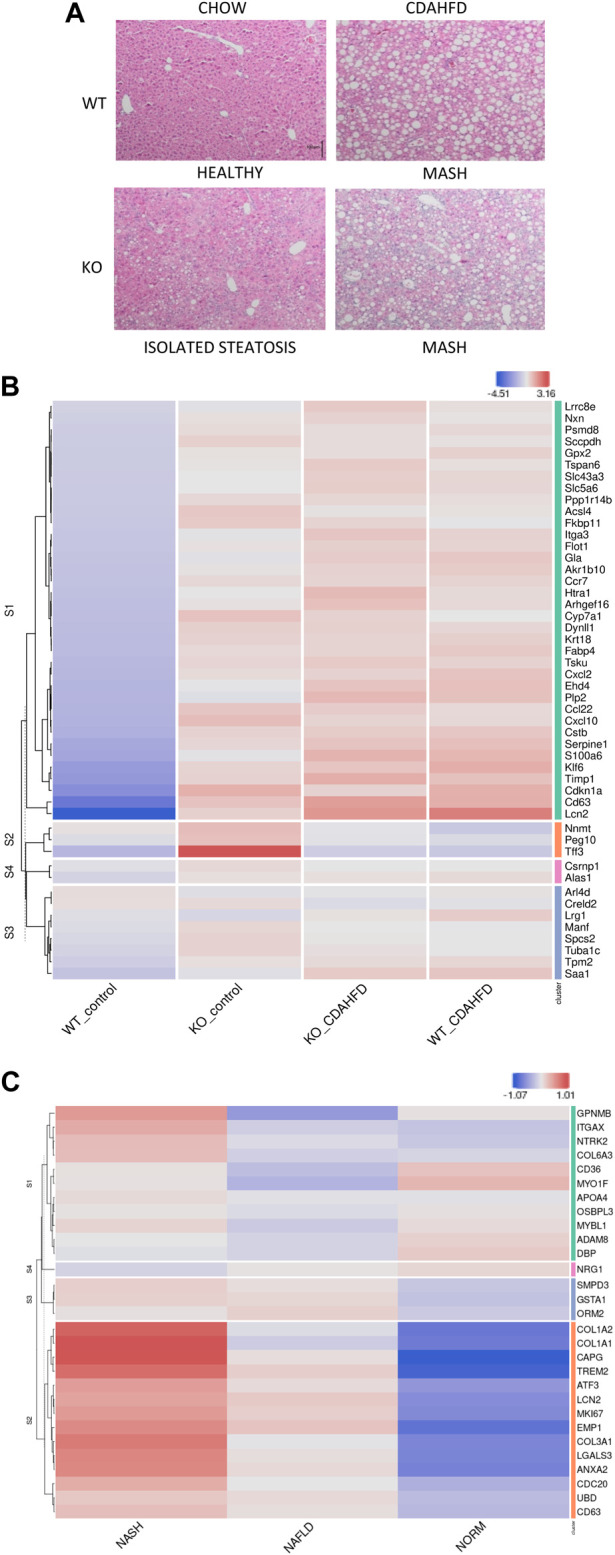
**(A)** H&E staining of liver sections of WT and hepatocyte PPARα KO mice after following 6-week chow or CDAHFD (scaling bar: 100μm) **(B)** Heatmap representation of MASLD signature in KO or WT mice after 6-week chow or CDAHFD (*n* = 3). **(C)** Heatmap representation of PPARα KO signature in MASLD (NAFLD) and MASH (NASH) patients of the GEO dataset GSE126848.

In line with reduced PPARα expression reported in MASLD/MASH patients, both the qPCR and western blot also reveal decreased PPARα expression in the WT mice following 6-week CDAHFD diet (Francque et al., 2015). The latter suggests that a high fat diet may gradually decrease PPARα expression and as such progressively phenocopies the transcriptome signature of a genetic hepatocyte-specific PPARα KO model. Furthermore, liver sections were scored based on the Clinical Research Network and Steatosis-Activity-Fibrosis NASH scoring systems to assess the disease stage of the mice (Figure 2A; Supplementary Table S2) (Kleiner et al., 2005; Bedossa et al., 2012). These results show that 6-week CDAHFD in WT and PPAR KO mice, both result in MASH features including steatosis, ballooning, lobular inflammation and fibrosis, similar to liver histopathology in MASH patients. Interestingly, PPARα KO mice on a normal chow diet already reveal MASLD features such as accumulation of lipid droplets.

To further characterize whether these histopathological changes also correlate with a MASLD/MASH disease signature in patients, we cross compared our gene expression profiles with the publicly available MASLD/MASH transcriptome signature of liver biopsies of a patient cohort with varying degrees of MASLD (GSE126848) (Supplementary Table S3). Interestingly, the hepatocyte-specific PPARα KO mouse model on both a chow and 6-week CDAHFD diet as well as WT mice after a 6-week CDAHFD diet, reveal a similar hyperactivated MASLD/MASH transcriptome signature, which confirms the involvement of a PPARα loss of function and disrupted fatty acid metabolism in the MASLD/MASH disease aetiology (Figure 2B). Accordingly, the MASLD/MASH heatmap of GSE126848 also phenocopies the PPARα loss of function transcription signature of the hepatocyte-specific PPARα KO mouse model (Figure 2C).

### 3.2 Genetic and diet-induced PPARα loss of function trigger lipid metabolic stress by DNA hypermethylation of PPARα target genes

**FIGURE 3:**
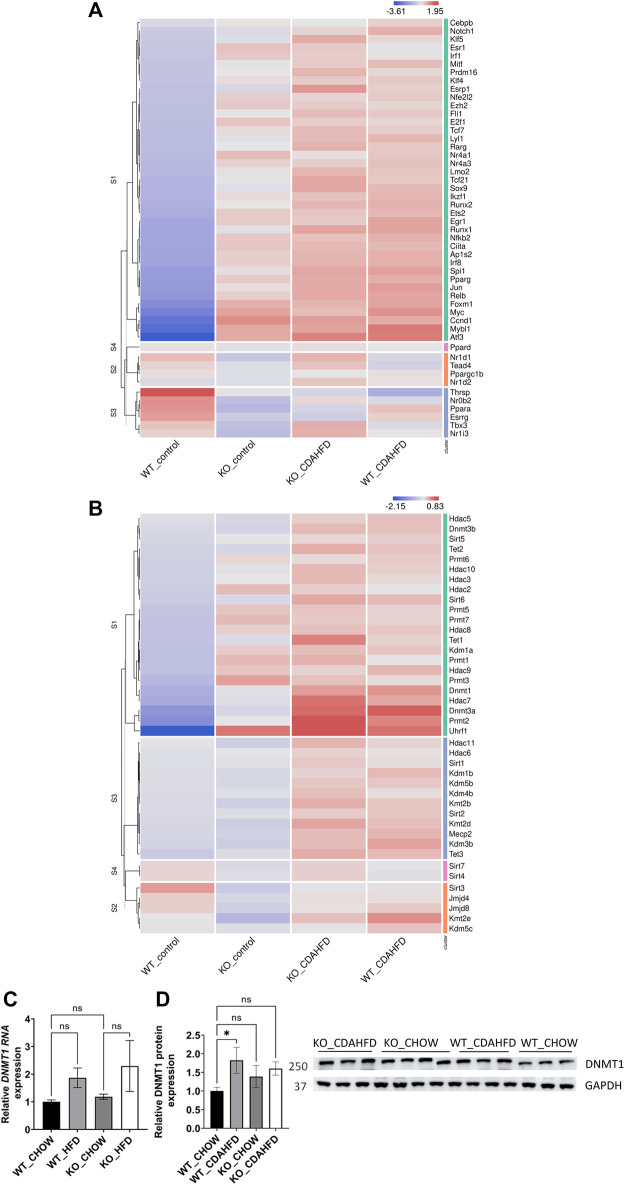
Heatmap representation of differentially expressed **(A)** transcription factors and nuclear receptors or **(B)** epigenetic writers-readers-eraser proteins in KO or WT mice after 6-week chow or CDAHFD (*n* = 3). **(C)** qPCR and **(D)** western blot detection and quantification of DNMT1 and GAPDH expression levels after 6-week chow or CDAHFD in WT and KO mice. Data are plotted as the mean ± s.d., *n* = 3 biologically independent replicates. (ns *p* > 0.05, *p < 0.05, **p < 0.01 ***p < 0.001)

Besides similarities in lipid metabolic gene expression changes between CDAHFD diet WT and chow/CDAHFD PPARα KO mice, genetically and diet-induced PPARα loss of function also regulate overlapping bile and fatty acid responsive transcription factors, nuclear receptors and epigenetic writer-reader-eraser proteins, including multiple DNA (hydroxy)methylating enzymes and DNA Methyl-binding factors (Figures 3A, B). For example, weakly increased RNA and protein expression levels of DNMT1 can be observed in PPARα KO and CDAHFD diet conditions (Figures 3C, D). Remarkably, Homer motif analysis revealed that several of these differentially expressed transcription factors and DNA Methyl-binding proteins themselves contain PPARα binding motifs (PPRE) motifs. Since these proteins can directly regulate epigenetic enzymes, these results suggest that epigenetic enzyme expression and activity too might be under lipidomic PPARα control (Supplementary Table S5).

**FIGURE 4:**
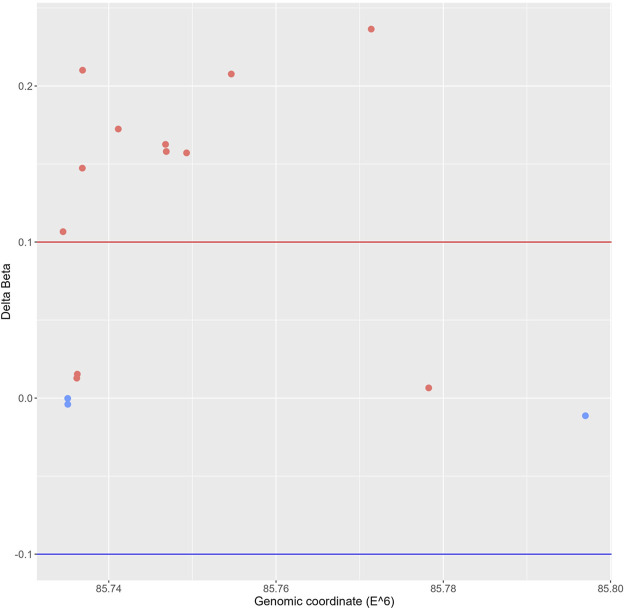
Genomic scatterplot of delta beta values of differently methylated probes in the promotor region of the PPARα gene in the WT group on a 6-week CDAHFD compared to the WT mice on 6-week chow diet.

**FIGURE 5:**
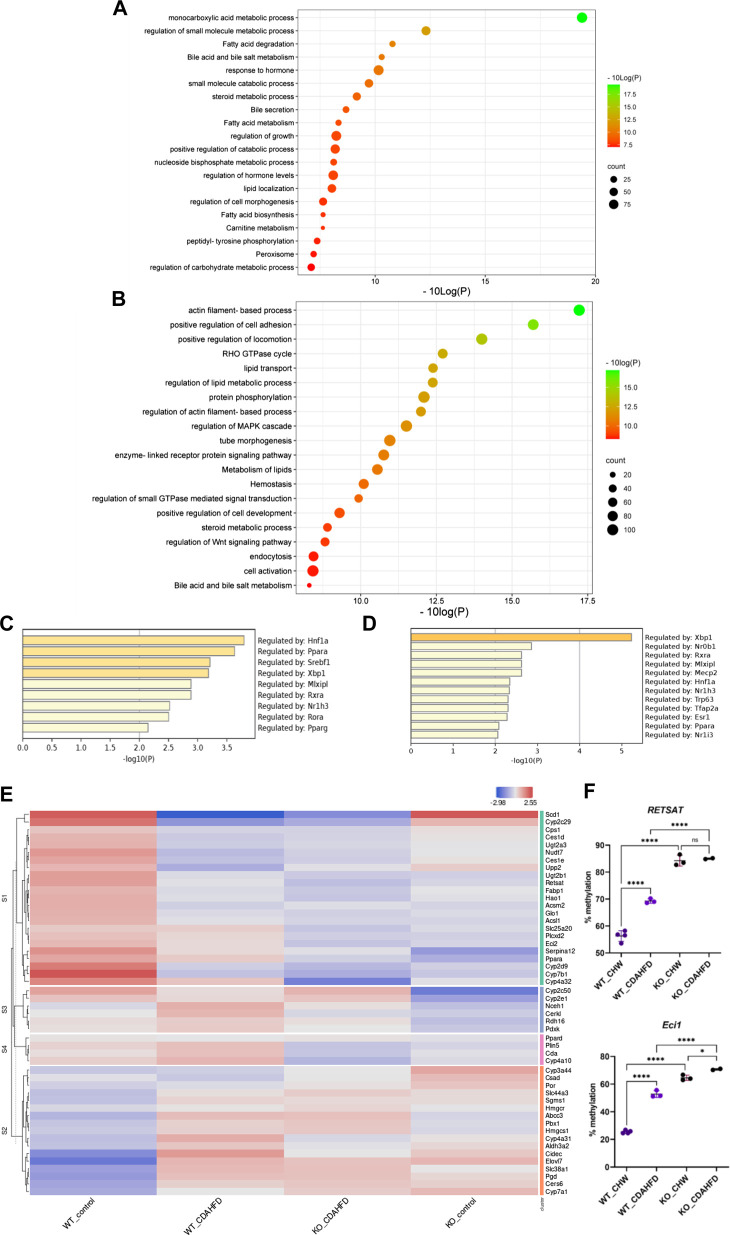
Metascape pathway analysis of differently methylated genes in **(A)** KO mice compared to WT mice on a chow diet (FDR<0.05; DB > 0.15) and **(B)** WT mice on a CDAHFD compared to a chow diet (FDR<0.05; DB > 0.25). TRRUST transcription factor analysis of differently hypermethylated genes in **(C)** KO mice compared to WT mice on a chow diet (FDR<0.05; DB > 0.15) **(D)** WT mice on a CDAHFD compared to a chow diet (FDR<0.05; DB > 0.25). **(E)** Heatmap representation of the expression of genes involved in lipid or bile acid metabolic pathways that are hypermethylated in WT mice on a CDAHFD and KO mice on a control chow diet compared to WT mice on a control chow diet. **(F)** Pyrosequencing validation of two PPARα target genes (ns *p* > 0.05, *p < 0.05, ****p < 0.0001).

Since altered DNA methylation has been identified as a key determinant of MASLD pathogenesis (Li et al., 2018; Hyun and Jung, 2020; Lai et al., 2020; Bu et al., 2022; Kuramoto et al., 2022; Melton et al., 2023), we next applied Infinium mouse methylation Beadchip array studies to map genome-wide DNA methylation changes in liver biopsies of WT and hepatocyte-specific PPARα KO mice, following 6-week chow or CDAHFD diet. As shown in Figure 4, the WT mice on a CDAHFD show predominant hypermethylation of the promotor region of PPARα compared to the WT mice on a chow diet, which could explain the gradual silencing of PPARα expression following a 6-week CDAHFD diet. Besides, both genetically and diet-induced PPARα loss of function trigger massive -partially redundant- DNA methylation changes in genes involved in fatty acid and bile acid metabolism, nuclear hormone (steroid) receptor and inflammatory cytokine pathways, according to Metascape enrichment analysis (Zhou et al., 2019) (Figures 5A, B). Remarkably, TRRUST motif analysis of hypermethylated genes in PPARα KO mice shows highly significant enrichment of PPRE (Figure 5C), which is still significantly enriched in WT CDAHFD mice which only partially express PPARα protein (Figure 5D). Along the same line, the cross-comparison of a list of PPARα target genes (Supplementary Table S4) with our lists of differentially methylated genes, identified various common hypermethylated target genes in the PPARα KO mice, following chow or CDAHFD diet, whereas CDAHFD diet in WT liver cells with partially decreased PPARα expression shows a mixed hypo/hypermethylation pattern (Supplementary Figure S2). Although most of the selected hypermethylated genes involved in fatty acid or bile acid metabolism, including PPARα metabolic target genes, are downregulated in the hepatocyte-specific PPARα KO mice and WT mice on a CDAHFD with a partial expression of PPARα, few genes are upregulated (Figure 5E). Remarkably, further validation on protein level showed that proteins involved in mitochondrial lipid uptake (CPT1A) are significantly upregulated by the loss of PPARα function, while lipid catabolic proteins involved in lipid or bile acid catabolism are downregulated (CYP7B1 and ACSM2). The latter emphasizes the dual epigenetic regulation of lipid metabolic genes via PPARα (Supplementary Figure S3). Of special note, bisulfite converted DNA assay does not allow discrimination between DNA methylation and hydroxymethylation changes that have been associated with gene silencing and gene activation responses respectively. Indeed, PPARα regulatory functions have recently been described for both DNA methylation as well as hydroxymethylation and may need a more detailed in-depth molecular investigation (Theys et al., 2022).

To further validate our epic array hypermethylation data, we applied bisulfite pyrosequencing of PPARα target genes *RETSAT* and *Eci1,* which are according to the EPIC data both hypermethylated by a diet-induced or genetic KO of PPARα (delta beta KO_chow vs. WT_chow: 0,48-0,29; KO_CDAHFD vs. WT_CDAHFD: 0,17-0,14; WT_CDAHFD vs. WT_chow: 0,31 -0,18). Moreover, these genes are involved in retinol metabolism and beta oxidation respectively and thereby control lipid metabolism (Pang et al., 2017; Bramhecha et al., 2022). As shown in Figure 5F relative DNA methylation is strongly increased when PPARα is knocked out, or modestly increased upon CDAHFD diet in WT mice with partially decreased PPARα expression. These results suggest that PPARα targeting of lipid metabolic genes may be essential to protect against epigenetic DNA methylation modifications.

**FIGURE 6:**
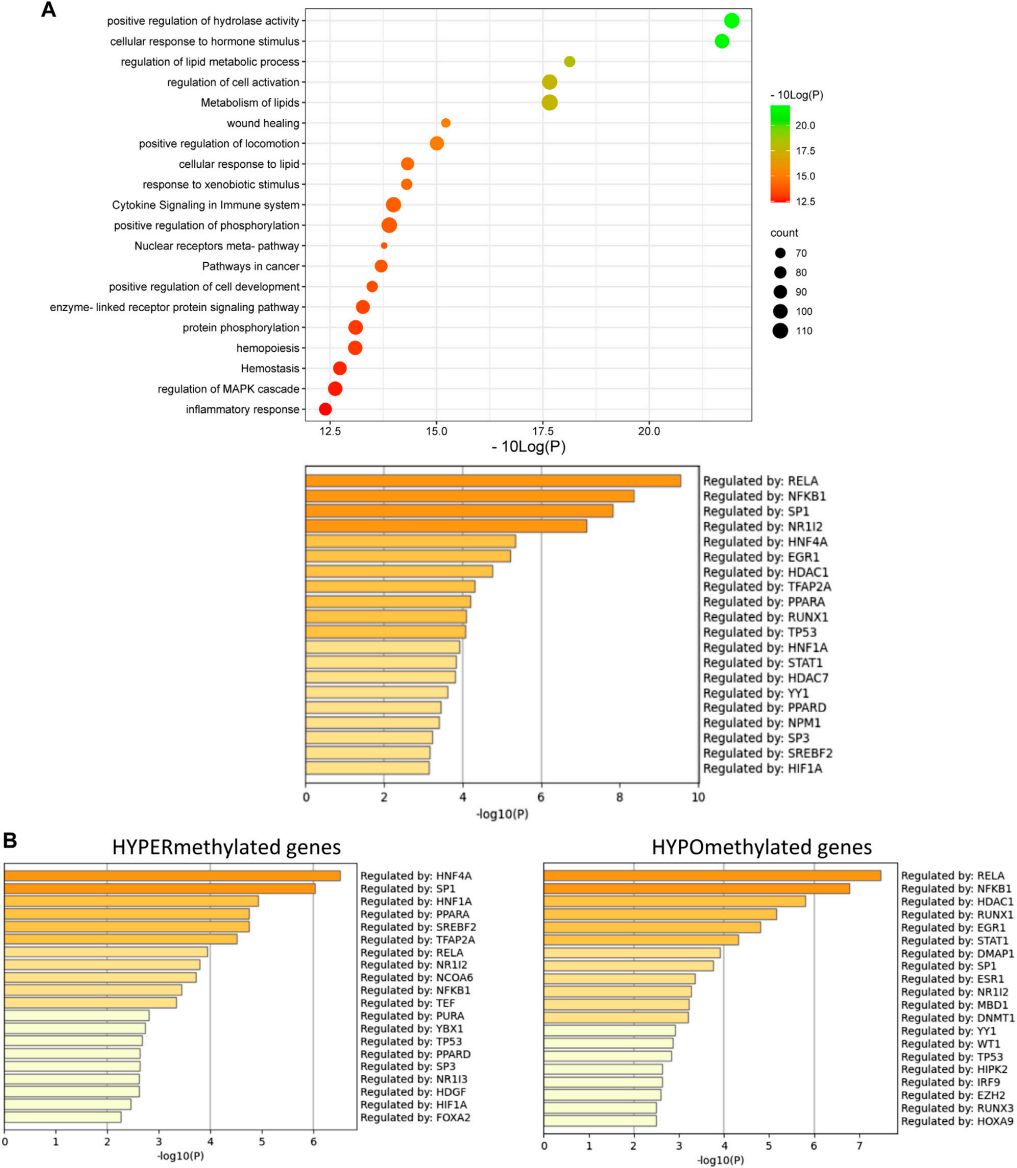
**(A)** Metascape pathway analysis (top) and TRRUST transcription factor analysis (bottom) of significant differently methylated genes in MASH patients with advanced fibrosis versus healthy individuals **(B)** TRRUST transcription factor analysis of significant hypermethylated and hypomethylated genes in MASH patients with advanced fibrosis versus healthy individuals. (*p*-value < 0.05; DB < |0.3|).

Furthermore, these results could also be confirmed in Epic Beadchip DNA methylation array data of MASLD liver biopsies of MASH patients (GSE241366) with advanced fibrosis in comparison to healthy tissue controls, which reveal similar enrichment of differentially methylated genes involved in lipid metabolism (fatty acid, bile acid) with PPARα as one of the top enriched TF motifs in general (Figure 6A) and especially of the hypermethylated genes (*p*-value < 0.05) (Figure 6B). Altogether this suggests that PPARα protects against epigenetic DNA (hyper)methylation of lipid metabolic genes involved in the progression of MASLD.

Of special note, in line with the fact that PPARα can also indirectly regulate genes via transrepression of other bound transcription factors such as NFκB (Delerive et al., 1999; Zúñiga et al., 2011; Tayyeb et al., 2020), we also observe multiple epigenetic changes of NFκB-driven (NFκB1, RelA, RelB) inflammatory target genes in the mouse/patient samples, which may further contribute to lipid-inflammation tissue damage in MASLD.

### 3.3 Genetic and diet-induced PPARα loss of function triggers epigenetic transition from lipid metabolic homeostasis to lipotoxic ferroptosis and pyroptosis in MASLD

Lipotoxic hepatocyte injury is a primary event in MASH, characterized by excess triglyceride accumulation stored as lipid droplets in the cytosol of hepatocytes, which is deemed the first stage of MASLD. Hepatic steatosis may further develop into MASH, fibrosis, cirrhosis and eventually hepatocellular carcinoma without timely interventions. Recent evidence suggests that hepatic ferroptosis and pyroptosis play an important role in this lipotoxic pathological progression of MASLD (Tsurusaki et al., 2019; Gaul et al., 2021; Zhang et al., 2021; Knorr et al., 2022).

Ferroptosis, a recently recognized nonapoptotic form of regulated cell death that is characterized by iron-dependent lipid peroxidation, was recently confirmed to be the initial cell death process that triggers MASH (Dixon et al., 2012; Tsurusaki et al., 2019; Zhang et al., 2021). Besides, new results identify hepatocyte pyroptosis and release of NOD-like receptor family pyrin domain containing 3 (NLRP3) inflammasome components as an additional mechanism to propagate liver injury and liver fibrosis development in MASH progression (Csak et al., 2011; Wree et al., 2014a; Beie et al., 2018; Wu et al., 2019). As the liver is a “first pass” organ, continually challenged with diverse microbial particles from the intestine as well as endogenous metabolic stress signals (fatty acid, bile acid), hepatocytes are capable of undergoing NLRP3-mediated pyroptotic cell death and release extracellular NLRP3 inflammasome complexes into the extracellular space. These extracellular inflammasomes can be internalized by hepatic stellate cells leading to their activation and subsequent liver fibrogenesis (Kisseleva and Brenner, 2007; Watanabe et al., 2009; Wree et al., 2014b; Wu et al., 2019; Knorr et al., 2022).

**FIGURE 7:**
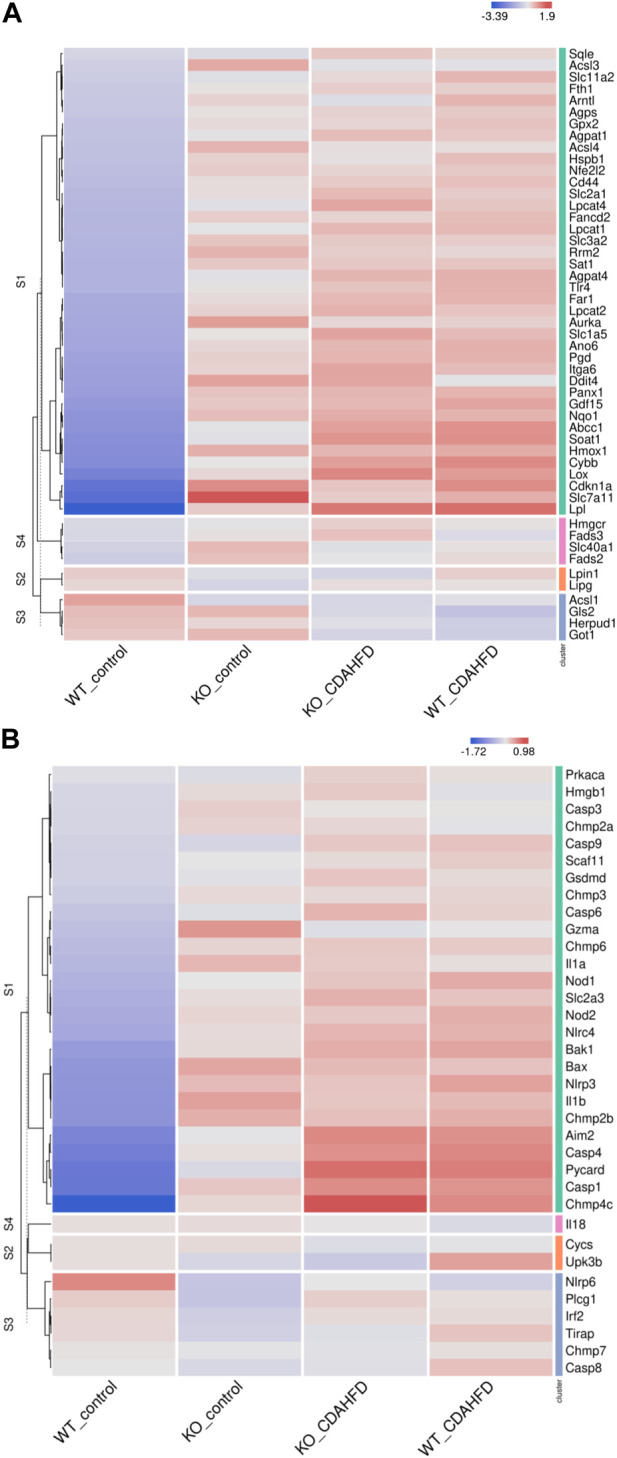
Heatmap representation of **(A)** ferroptosis and **(B)** pyroptosis signature in WT or PPARα KO mice on a 6-week chow or CDAHFD.

To evaluate whether PPARα loss may impact ferroptosis/pyroptosis pathways in MASLD/MASH, we next performed a cross comparison of our gene expression mouse data with publicly available ferroptosis/pyroptosis RNAseq based transcriptome datasets (Hassannia et al., 2018; Ye et al., 2021; De Backer et al., 2022; Deng et al., 2022; Wang et al., 2022; Zuo et al., 2022; Zhou et al., 2023). Remarkably, both genetic and diet-induced PPARα loss reveal strong hyperactivation of lipotoxic ferroptosis/pyroptosis signatures (Figure 7). Indeed, further protein level analysis confirmed a significant upregulation of the nuclear factor E2 related factor 2 (Nrf2/NFE2L2), a key regulator of the ferroptosis (Hassannia et al., 2018; Pan et al., 2021) and pyroptosis (Hurtado-Navarro et al., 2022) pathways, in PPARα KO mice on chow diet and WT or KO mice on CDAHFD diet. Moreover, a significant upregulation of malondialdehyde (MDA), which represents increased lipid peroxidation, was found under a CDAHFD in both the KO and WT mice, in line with observations in MASLD/MASH patient samples (Zhang et al., 2021). Furthermore, protein validation of Caspase 1 and NLRP3 showed that a genetic and diet-induced PPARα loss induce an upregulation of NLRP3. However Caspase 1 is not further cleaved in its catalytic domains p10 and p20, indicating that PPARα loss increases sensitivity for pyroptosis without inducing further pyroptotic cell death. Of special note, when cross comparing differentially methylated target genes of advanced MASH (versus healthy liver biopsies), with both ferroptosis/pyroptosis genesets, we could identify various novel epigenetic biomarkers of ferroptosis/pyroptosis lipotoxicity (Figure 9; Supplementary Table S6).

Altogether, these results suggest that PPARα function is essential to prevent the epigenetic transition from lipid homeostasis to MASH/MASLD lipotoxicity. In addition, epigenetic ferroptosis/pyroptosis biomarkers might hold promise as new precision medicine tools in MASLD/MASH disease management and patient stratification of lipotoxic liver damage.

## 4 Discussion

The nuclear receptor PPARα is a critical regulator of lipid metabolism and MASLD progression. Surprisingly, PPARα ligands have only shown limited therapeutic benefits against MASLD in (pre)clinical trial studies (Lange et al., 2022). Of special note, recent evidence suggests a possible involvement of epigenetic silencing mechanisms in PPARα functions in MASLD progression which may counteract therapeutic actions of PPAR ligands (Theys et al., 2022). In this respect, to further characterise reciprocal crosstalk of epigenetic regulatory mechanisms with PPARα functions in lipid metabolism and MASLD progression, we have cross compared DNA methylation and gene expression patterns of chow or CDAHFD hepatocyte-specific PPARα KO mice versus liver biopsies of MASLD/MASH patient samples.

Upon comparing gene expression changes of liver biopsies of hepatocyte-specific PPARα KO mice versus WT mice following 6-week chow diet, or WT mice following 6-week CDAHFD, we observed strong similarities in transcriptome signatures with the CDAHFD WT mice. This reveals that CDAHFD phenocopies to some extent a genetic KO of PPARα liver functions. QPCR and western blot analysis of PPARα expression indeed confirmed a lack of PPARα protein expression, whereas CDAHFD revealed significantly decreased PPARα expression as compared to chow diet fed WT mice. Moreover, in line with the gene expression profiles in mice, publicly available gene expression datasets of liver biopsies of MASLD/MASH patients show high similarities with the PPARα KO transcriptome signature, which reveals loss of PPARα function in these patients. This is in line with reduced PPARα expression levels which have been observed in MASH/MASLD patients (Francque et al., 2015). Reciprocally, we observed that transcriptome profiles of CDAHFD fed WT and PPARα KO mice also show high similarity to a MASLD/MASH patient signature, which suggests that CDAHFD fed WT and PPARα KO mice are clinically relevant mouse models for molecular biochemical investigation of MASH/MASLD disease. In line with these results, histological staining of liver biopsies confirmed increased frequency of lipid droplets in chow fed PPARα KO mice (stage 2, isolated steatosis), as well as inflammatory ballooning and fibrosis properties in CDAHFD fed PPARα KO/WT mice (stage 3, MASH). These results also confirm the data of Matsumoto et al. (2013) who has previously shown that a CDAHFD can induce MASH with fibrosis in mice in 6-week time, which is relatively fast compared to classical HFD used to induce MASLD in mice. By studying this mouse model, we were able to functionally characterize epigenetic driver and passenger functions of PPARα in lipid metabolism in relation to MASLD/MASH, which has not been addressed before.

Remarkably, diet and genetic PPARα knockout mice elicit similar transcriptional activation of multiple transcription factors, steroid hormone receptors and epigenetic factors involved in metabolic stress responses in the liver (Figures 3A, B). This is not completely unexpected, since loss of hepatocyte PPARα function results in loss of lipid metabolism homeostasis due to impaired fatty acid, bile acid and amino acid catabolic processes (Figure 1D) which results in major changes in the lipidome composition (Régnier et al., 2020). Accordingly, multiple compensation mechanisms of lipid sensing transcription factors and nuclear hormone receptors (Myc, NR4A1, NR4A3, PPARδ/γ, E2F1, PPARGC1B, Nrf2/NFE2L2, TCF21) are activated to mitigate lipidomic stress and to alleviate mitochondrial metabolic stress (Walczak and Tontonoz, 2002; Zirath et al., 2013; Denechaud et al., 2015; Sharma et al., 2018; Yan et al., 2018; Li et al., 2020; Cariello et al., 2021; Mohs et al., 2021; Ni et al., 2023). Similarly, expression of various epigenetic factors (DNMT, TET, SIRT, HDAC, Uhrf1) changes upon lipid metabolic inflammatory stress (Kemper et al., 2013; Ding et al., 2017; Keleher et al., 2018; Wang et al., 2020; Byrnes et al., 2022; Hou et al., 2022; Dong, 2023; Sukur et al., 2023), some of which contain PPRE motifs in their gene promoters (Supplementary Table S5). Although the full mechanism has not been resolved, it appears that there is reciprocal regulation between PPARα protein levels versus expression of DNMT, TET and other DNA-methyl binding proteins such as Uhrf1 (Unoki et al., 2004; Ashraf et al., 2017; Ren, 2022). Moreover, besides transcriptional control mechanisms, PPARα-dependent β-oxidation also promotes (mitochondrial) protein hyperacetylation via increased acetyl-coA production, which can change protein function, localisation, interaction and/or stability (Pougovkina et al., 2014).

**FIGURE 8:**
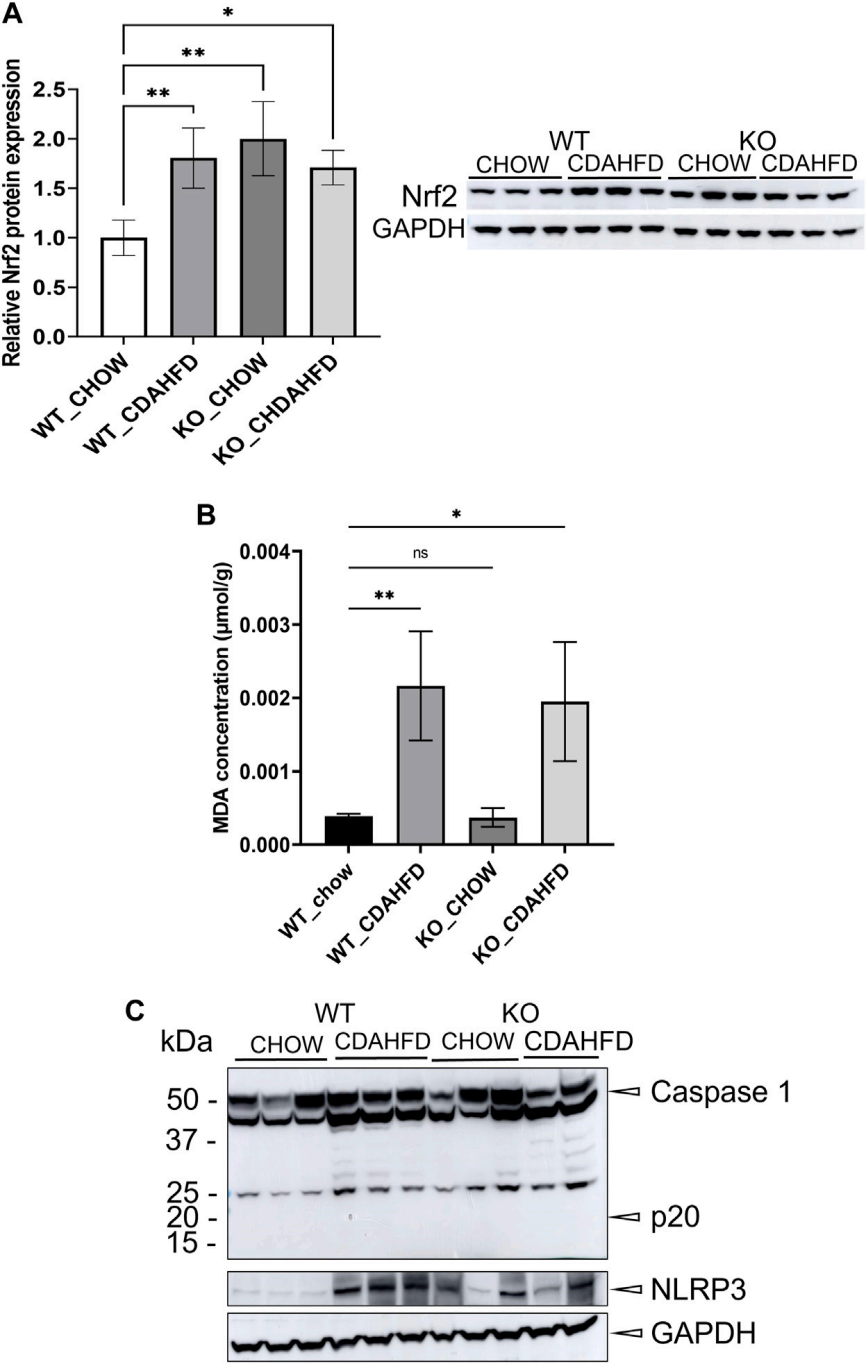
**(A)** Western blot detection and quantification of NRF2 and GAPDH protein expression levels and **(B)** MDA concentration in liver samples of WT and KO mice after a 6-week chow or CDAHFD **(C)** Western blot detection of Caspase 1, NLRP3 and GAPDH in WT and KO mice after a 6-week chow or CDAHFD. Data are plotted as the mean ± s.d (ns *p* > 0.05, *p < 0.05, **p < 0.01 ***p < 0.001).

**FIGURE 9:**
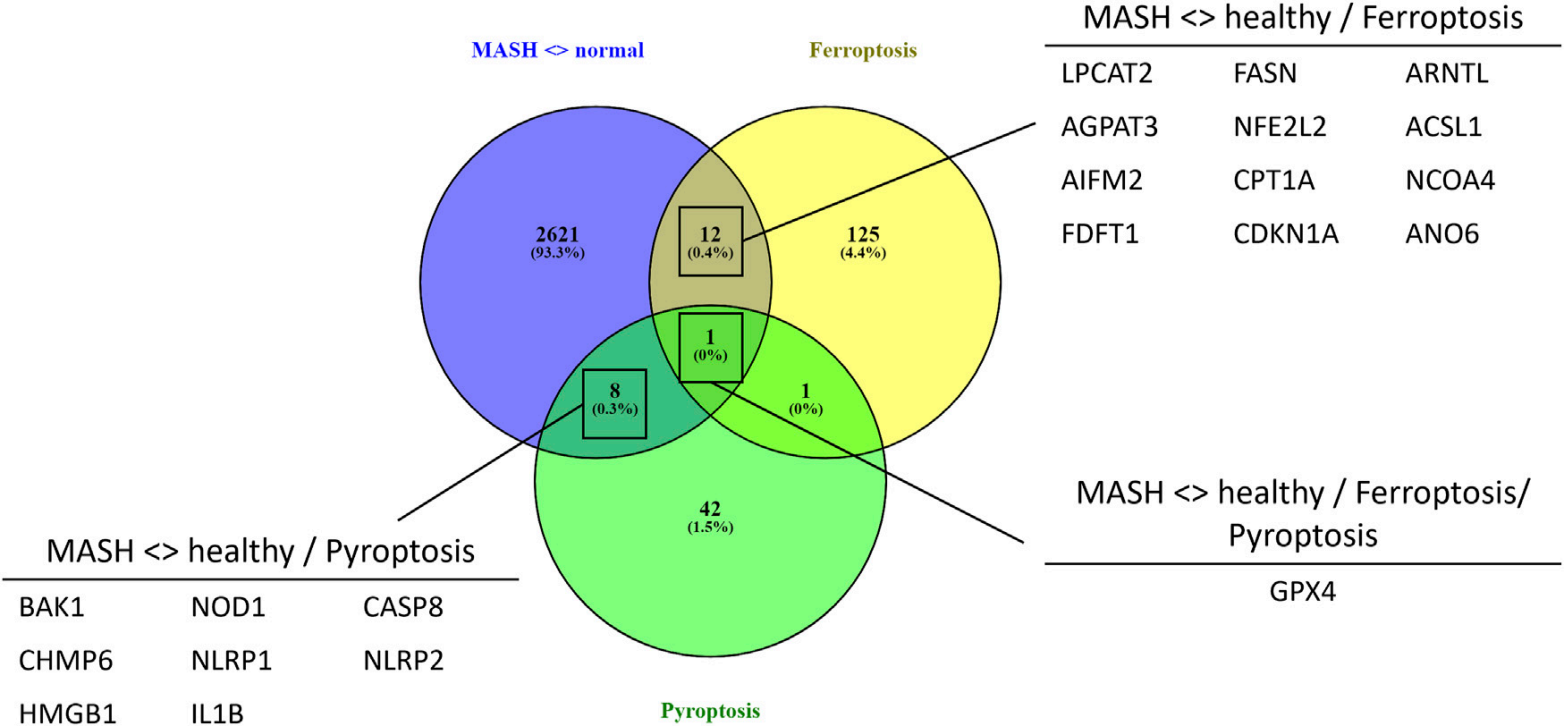
Venn diagram showing the overlap between significant differentially methylated genes in MASH patients compared to normal liver controls and a ferroptosis or pyroptosis signature (*p*-value <0.05, DB>|0.3|).

Not surprisingly, diet and genetic loss of PPARα hepatocyte function trigger massive DNA methylation changes of multiple genes associated with fatty acid, bile acid and steroid hormone receptor pathways, including PPARα, which changes the expression of multiple lipid metabolic genes (Figure 5). Since bisulfite sequencing does not discriminate between DNA methylation and hydroxymethylation changes, epigenetic changes trigger mixed metabolic gene silencing-activation effects involved in lipid metabolism and MASLD (RETSAT, FABP1, Eci1/2, Cyp7a1) (Lyall et al., 2016; Chiang, 2017; Heidenreich et al., 2017; Mukai et al., 2017; Lyall et al., 2020; Rom et al., 2020). Moreover, epigenetic changes following loss of PPARα functions seem to fail to mitigate liver metabolic dysfunctions (autophagy, mitophagy, lipophagy), since downstream gene expression profiles and key regulatory proteins of ferroptosis and pyroptosis lipotoxicity pathways are highly enriched (Figures 7, 8). Indeed, liver overload of fatty acid and bile acid metabolites promotes lipid peroxidation ferroptosis damage and sensitizes for inflammation-induced steatosis-pyroptosis (Figure 8), which can finally trigger liver fibrosis and cirrhosis or hepatocellular carcinoma (Tsurusaki et al., 2019; Wu et al., 2019; Gaul et al., 2021; Wu et al., 2021; Zhang et al., 2021; Chen et al., 2022; Knorr et al., 2022). Accordingly, we identified various epigenetic changes in ferroptosis-pyroptosis target genes in liver biopsies of late-stage MASLD/MASH patients, which could hold promise as novel stochastic biomarkers of lipid-related inflammatory liver damage (Figure 9; Supplementary Table S6), besides fibrosis stage (Sokolowska et al., 2022; Sun et al., 2022) or epigenetic clock age (Loomba et al., 2018; Li et al., 2022). Interestingly, PPARα was recently shown to protect against liver ferroptosis and might further suppress pathological progression into liver pyroptosis-fibrosis-cirrhosis (Grabacka et al., 2021; Xing et al., 2022; Qiu et al., 2023; Xiao et al., 2023). Of special note, PPARα DNA-binding was demonstrated to the Gpx4 promoter by ChIP experiments (Xing et al., 2022). These findings suggest that ferroptosis inhibitors and epigenetic drug combination therapies with PPAR ligands could hold promise to treat MASLD/MASH.

In conclusion, we demonstrate that loss of PPARα function promotes epigenetic dysregulation of lipid homeostasis, driving ferroptosis and pyroptosis lipotoxicity in MASLD. Of special note, loss of function of a single lipid metabolic PPARα hub seems to cause a lipidomic shockwave of gene expression changes of lipid sensing transcription factors and epigenetic enzymes, which fail to mitigate lipid metabolic stress and trigger epigenetic transition towards lipid hepatotoxicity driving fibrosis. This may explain why monotargeted therapeutic strategies in MASLD/MASH may not be effective to “cure” the multi-factorial nature of MASLD involving genetic predisposition, environmental factors (lifestyle, diet), insulin resistance, disordered lipid metabolism, mitochondrial dysfunction, lipotoxicity, hyperinflammation, oxidative stress, etc. This urges for applying integrative multi-omics systems biology approaches (incorporating data on genetic variants, epigenetic phenomena (i.e., DNA methylation, histone modifications and long non-coding RNA affecting gene expression), gut microbiota dysbiosis, and metabolomics/lipidomic fingerprints) to gain a deeper understanding of the molecular and physiological processes underlying MASLD pathogenesis and phenotype heterogeneity, as well as facilitating the further identification of lipidome-associated epigenetic biomarkers of disease progression and therapeutic targets for the implementation of tailored nutritional strategies (Buzzetti et al., 2016; Soltis et al., 2017; Fang et al., 2018; Ore and Akinloye, 2021; Minami et al., 2023). In this respect, besides pharmaceutical combination therapies, diet interventions and herbal phytomedicinal therapies may also have a role to play in the treatment of MASLD, due to their numerous bioactive constituents and the multiple pharmacological actions they exhibit (Gao et al., 2020; Ore and Akinloye, 2021; Cheng et al., 2022). Finally, to capture a full understanding of adverse MASLD epigenome dysregulation, it will be mandatory to also integrate the complex epi-lipidomic post-translational modification landscape of transcription factors, histones and epigenetic modifiers which control the lipid metabolic network signalling activities in MASLD progression (Amado et al., 2014; Kebede et al., 2017; Sabari et al., 2017; Nieborak and Schneider, 2018; Bartke and Schneider, 2020; Dai et al., 2020; Arima et al., 2021; Guerra et al., 2022).

## Data Availability

The datasets presented in this study can be found in online repositories. The names of the repository/repositories and accession number(s) can be found below: https://www.ncbi.nlm.nih.gov/, GSE238173 https://www.ncbi.nlm.nih.gov/, GSE238201.

## Supplementary material

SUPPLEMENTARY TABLE S1 Overview of qPCR, PCR and pyrosequencing primers used in this study. (b = biotin tag)

SUPPLEMENTARY TABLE S2 Full CRN and SAF scoring of the mice used for RNA sequencing and Beadchip analysis.
