# Supplementary material for: Loss of PPARα function promotes epigenetic dysregulation of lipid homeostasis driving ferroptosis and pyroptosis lipotoxicity in metabolic dysfunction associated Steatotic liver disease (MASLD)
Source: Front Mol Med. 2024 Jan 8;3:1283170. doi: 10.3389/fmmed.2023.1283170 (PMC11285560; doi:10.3389/fmmed.2023.1283170)
Supplement: Supplementary file 1 [file Table2.DOCX]

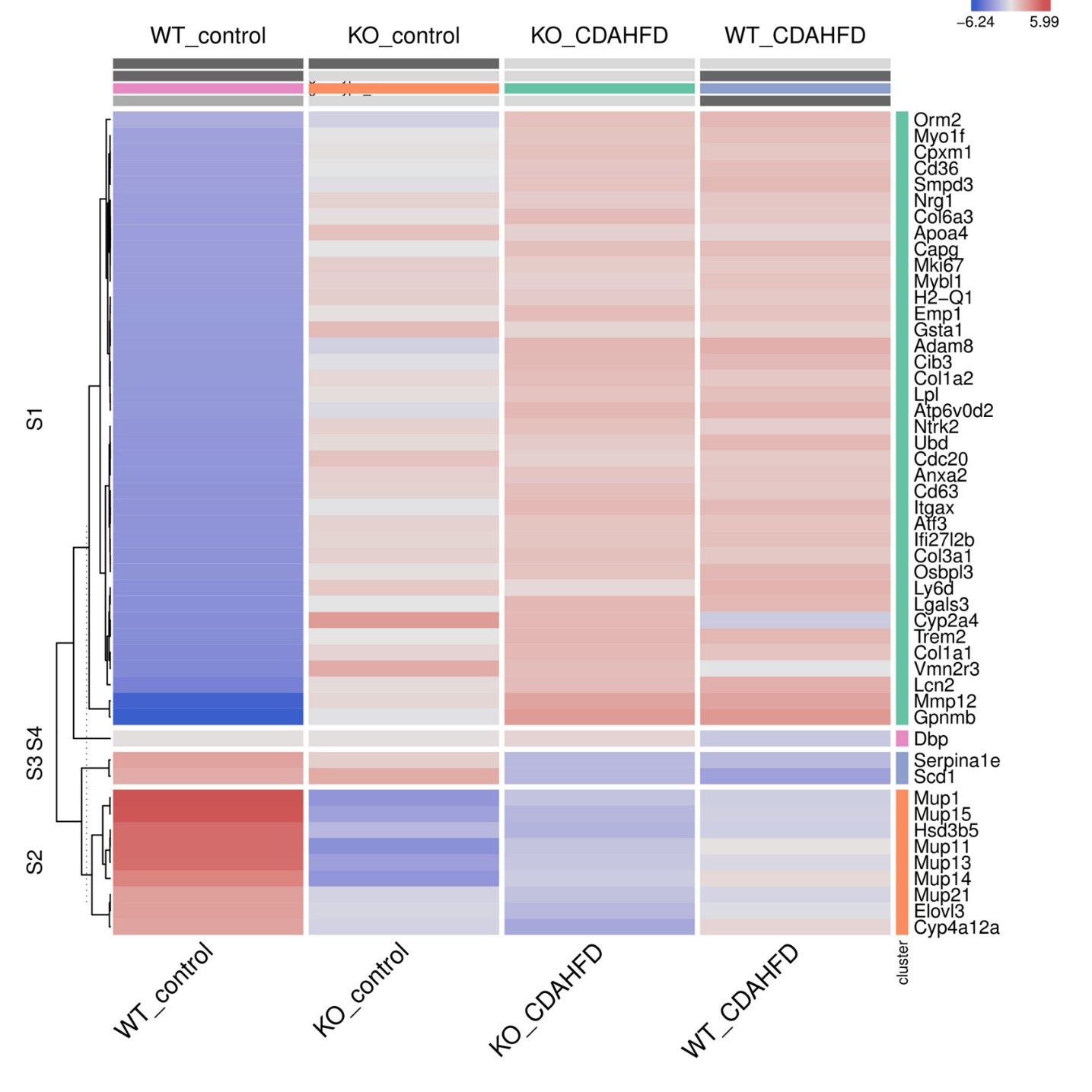


**Supplementary figure 1: Heatmap representation of differentially expressed genes in WT or PPARα KO mice on a 6-week chow or CDAHFD showing a similar expression in genes induced by a diet induced downregulation or KO of PPARα.**


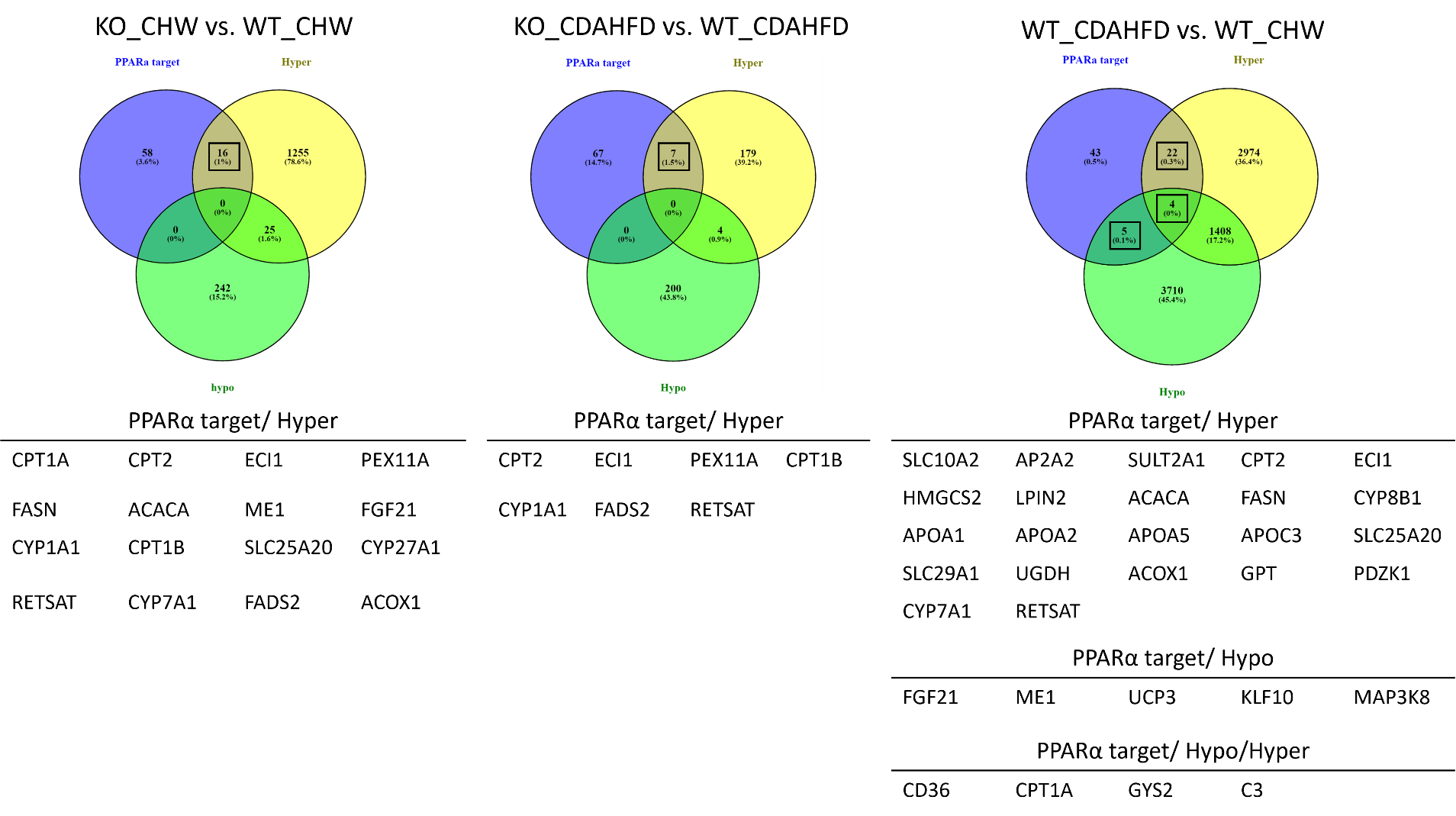


**Supplementary figure 2: Venn diagram showing the overlap between significantly hypermethylated (hyper), hypomethylated (hypo) probes and a list of PPARα target genes (PPARa target) in KO mice versus WT mice on a chow diet (left; FDR<0.05; DB>|0.15|), KO mice versus WT mice on a CDAHFD (middle; FDR<0.05; DB>|0.1|) and WT mice on a CDAHFD versus chow diet (right; FDR<0.05; DB>|0.15|).**


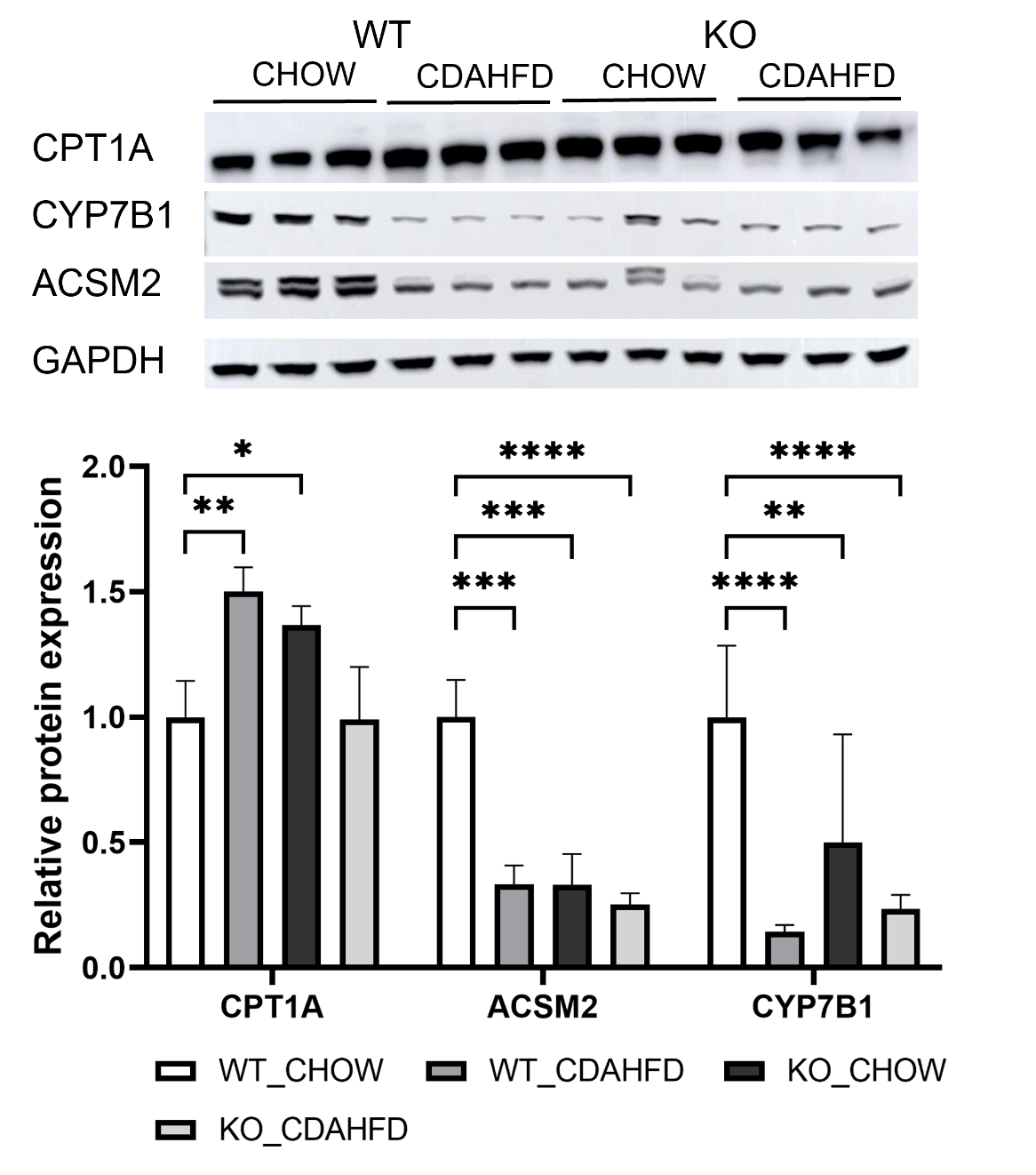


**Supplementary figure 3: Western blot detection and relative quantification of CPT1A, ACSM2, CYP7B1 and normalized against GAPDH protein expression levels in WT or PPARα KO mice on a 6-week chow diet or CDAHFD (ns p > 0.05, *p < 0.05, **p < 0.01 ***p < 0.001, ****p < 0.0001).**
